# A Community-Based Study of Factors Associated with Continuing Transmission of Lymphatic Filariasis in Leogane, Haiti

**DOI:** 10.1371/journal.pntd.0000640

**Published:** 2010-03-23

**Authors:** Alexis Boyd, Kimberly Y. Won, Shannon K. McClintock, Catherine V. Donovan, Sandra J. Laney, Steven A. Williams, Nils Pilotte, Thomas G. Streit, Madsen V. E. Beau de Rochars, Patrick J. Lammie

**Affiliations:** 1 Division of Parasitic Diseases, Centers for Disease Control and Prevention, Atlanta, Georgia, United States of America; 2 Department of Biological Sciences, University of Notre Dame, Notre Dame, Indiana, United States of America; 3 Department of Biological Sciences, Smith College, Northampton, Massachusetts, United States of America; 4 Hopital Ste. Croix, Leogane, Haiti; New York Blood Center, United States of America

## Abstract

Seven rounds of mass drug administration (MDA) have been administered in Leogane, Haiti, an area hyperendemic for lymphatic filariasis (LF). Sentinel site surveys showed that the prevalence of microfilaremia was reduced to <1% from levels as high as 15.5%, suggesting that transmission had been reduced. A separate 30-cluster survey of 2- to 4-year-old children was conducted to determine if MDA interrupted transmission. Antigen and antifilarial antibody prevalence were 14.3% and 19.7%, respectively. Follow-up surveys were done in 6 villages, including those selected for the cluster survey, to assess risk factors related to continued LF transmission and to pinpoint hotspots of transmission. One hundred houses were mapped in each village using GPS-enabled PDAs, and then 30 houses and 10 alternates were chosen for testing. All individuals in selected houses were asked to participate in a short survey about participation in MDA, history of residence in Leogane and general knowledge of LF. Survey teams returned to the houses at night to collect blood for antigen testing, microfilaremia and Bm14 antibody testing and collected mosquitoes from these communities in parallel. Antigen prevalence was highly variable among the 6 villages, with the highest being 38.2% (Dampus) and the lowest being 2.9% (Corail Lemaire); overall antigen prevalence was 18.5%. Initial cluster surveys of 2- to 4-year-old children were not related to community antigen prevalence. Nearest neighbor analysis found evidence of clustering of infection suggesting that LF infection was focal in distribution. Antigen prevalence among individuals who were systematically noncompliant with the MDAs, i.e. they had never participated, was significantly higher than among compliant individuals (p<0.05). A logistic regression model found that of the factors examined for association with infection, only noncompliance was significantly associated with infection. Thus, continuing transmission of LF seems to be linked to rates of systematic noncompliance.

## Author Summary

Lymphatic filariasis (LF) is a mosquito-borne parasitic disease that affects an estimated 120 million people worldwide with over 1 billion at risk for infection. LF is considered to be a leading cause of permanent disability worldwide due to the clinical manifestations of the disease. A global effort was established to eliminate LF by 2020 through interruption of transmission by annual mass administrations of anti-parasitic drugs. In Leogane, Haiti, seven rounds of drug administration have been administered and, though infection levels have dropped, transmission has not been interrupted. In this study the authors examined factors that could contribute to continuing transmission of LF in Haiti. Ongoing transmission was confirmed by high infection rates among young children. Infection was found to cluster at the household level within communities. The factor most associated with this transmission was systematic noncompliance with drug administration (i.e. never taking the medication). While increased health education and awareness campaigns may improve noncompliance, new tools and approaches may be needed to stop transmission of LF in Haiti. Understanding obstacles and solutions from the Haiti program could aid elimination programs in other countries.

## Introduction

Lymphatic filariasis (LF) is a mosquito-transmitted parasitic disease that is ranked by the World Health Organization (WHO) as a leading cause of permanent disability worldwide. LF affects an estimated 120 million people in 81 countries, with over 1 billion, or one-fifth of the world's population, at risk for filarial infection due to their exposure to infective larvae through the mosquito vector [1]. LF causes debilitating genital disease (hydrocele) in an estimated 25 million men and lymphedema or elephantiasis of the leg in 15 million people, mostly women [2].

The Global Programme to Eliminate Lymphatic Filariasis (GPELF) was established in 2000 with the goal of eliminating LF as a public health problem worldwide by 2020. The programme is centered on annual mass drug administrations (MDAs) which are based on the community-wide distribution of albendazole plus either diethylcarbamazine (DEC) or ivermectin to all those at risk in an endemic community. These drug combinations suppress the parasite in the blood, thereby reducing the transmission potential of the parasite, and also kill a broad spectrum of intestinal worms [3]. The WHO currently recommends mass treatment in settings where the prevalence of antigenemia is ≥1%. [4] For endemic countries, it is thought that 4–6 rounds of MDA, with at least 60–70% compliance, are adequate to interrupt transmission.

GPELF has scaled up impressively since it began. By the end of 2007, 48 countries had implemented elimination programs and approximately 570 million people had been treated. [1] In Haiti, the national program began in 2001 after a demonstration project in the Leogane commune started in 2000. The program conducted an initial LF infection survey by testing schoolchildren for the presence of filarial antigen in each of the 133 communes in the country. The results of this survey were compiled into an infection map [5]. Treatment of the hyperendemic communes (>10% antigenemia in children) has been the highest priority of the National Programme for Elimination of Lymphatic Filariasis (NPELF). Annual MDA in Haiti was gradually scaled up between 2000–2005 in all hyperendemic communes, except those areas where political instability in 2004–2005 threatened the safety of distribution staff (e.g. Port au Prince and Gonaives). Because of an interruption in funding there was no MDA in Haiti in 2006, but MDA resumed in 2007.

The current benchmark for success, as defined by the WHO, is a microfilaria prevalence of less than 1% in a community. Microfilaremia is indicative of the presence of motile larvae of the parasite (microfilariae) in the blood stream. If microfilaremia in a community is less than 1%, it is thought that transmission of the parasite cannot be sustained and there should be few or no incident infections. In order to measure the effect of the MDAs on transmission, and thus their effectiveness, surveys were conducted at sentinel sites around Leogane commune [6]. In 2005, the microfilaremia in Leogane was below 1%, reaching the WHO benchmark for successful interruption of transmission [7]. In these settings, current WHO guidelines recommend conducting cluster surveys of young children (2–4 years) to confirm the interruption of transmission and determine whether MDA can be halted [8]. In the absence of transmission and after at least 5 rounds of MDA, children under 5 years of age are expected to be antigen and antibody-negative. In August 2007 a survey was conducted of children 2–4 years of age, residing in Leogane. Blood samples for antigen and antibody testing were obtained. The results of the survey showed 14% antigen prevalence by ICT test and 19% antibody prevalence in children ages 2–4 years (n = 304) (Donovan et. al, unpublished data). These findings indicate that six rounds of MDA have not been sufficient to interrupt transmission in Leogane.

Although the WHO recommendations do not include monitoring of mosquito infection, information regarding the persistence of infection in the mosquito population is useful in determining whether transmission is ongoing. Persistence of filarial DNA in the mosquito population could be an indication that transmission has not been interrupted. The current study was designed to confirm continuing transmission of LF using blood and mosquito diagnostic tools and to analyze factors that may be contributing to the continued transmission of LF in Leogane, including population migration and systematic noncompliance.

## Methods

### Study Site

Surveys were conducted in Leogane, Haiti. Leogane is located 30 km west of the capital Port-au-Prince. Based on initial antigen prevalence as high as 50%, Leogane commune was considered to be highly endemic for LF. Seven rounds of MDA had been carried out prior to the present surveys for which data were collected between January 15 and February 19, 2008.

### Study Design

Villages representing six communities were selected from those that were surveyed in the August 2007 study. Four communities with two or more antigen positive children out of ten tested [Guinebeau (4 positive), Corail Lemaire (4 positive), Dampus (3 positive), and Leogane (2 positive)] and two communities with no antigen positive children (Santo and Dufort) were selected for this study. In each village, 100 houses were mapped using Personal Digital Assistants (PDAs) (Dell, Round Rock, Texas) equipped with GPS. Thirty houses and 10 alternates were chosen randomly by the PDA program for the study. Residents of selected households were interviewed using a standardized questionnaire and blood samples were collected at night by fingerprick. Due to low levels of literacy all participants were given explanations of the study and gave their verbal consent to be interviewed and bled for filarial testing. Consent was documented on the PDAs. For children under the age of 12 years, parents or guardians gave the consent. The study protocol was reviewed and approved by the Institutional Review Board at the Centers for Disease Control and Prevention and the Ethics Committee of Hopital Ste. Croix.

### GPS Mapping and Sampling

Two teams surveyed each of the communities. Details of the PDA and GPS programs are described elsewhere [9]. In Guinebeau, Corail Lemaire, Dampus and Leogane, mapping started at a house previously identified as having an antigen positive child, which was designated as the index house. This house was mapped but not considered for selection to participate in the study. In communities with no antigen positive children in the previous survey (Santo and Dufort), a house that was centrally located was randomly chosen. The two teams each mapped 50 houses in opposite directions from the index or centrally located house. The GPS data were synchronized and 30 households and 10 alternates were chosen by the GPS program to participate in the study. The 30 households were visited first and if the residents from any household declined to participate in the study, one of the alternate houses was chosen. An attempt was made to revisit households where no one was home before an alternate house was selected.

### Questionnaire

A short questionnaire was developed for the PDAs using Visual CE, as described elsewhere [9]. Each participant was asked to provide demographic information (age, sex, birthplace) that was recorded in the PDA. Individuals were asked questions regarding travel, general knowledge of lymphatic filariasis, compliance with previous MDAs and, if noncompliant, reasons for noncompliance. In order to determine travel history, participants were asked if they had left Leogane in the past, for how long and where they had traveled. For noncompliance, participants were asked if they had ever participated in an MDA, if they had participated in the previous MDA (2007) and the MDA before that (2005). Systematic noncompliance was defined as never having participated in an MDA. Unless otherwise stated, the term noncompliance refers to systematic noncompliance. Participants were asked if they had lived in Leogane their entire lives and if not, when they had moved to Leogane. These questions were used to determine migration into **Leogane.** Each completed survey was connected by the household variable to the GPS coordinates for that house. For children under 12 years of age, parents or guardians completed the questionnaire for the children. Individuals responding to the questionnaire were not prompted or coached in their responses.

### Blood Collection

Residents of households that had been selected for interviews were revisited in the evening (8–10 pm) and individuals older than three years of age were asked to provide blood samples. High-flow lancets (Becton Dickinson, Franklin Lakes, NJ) and EDTA coated blood collection tubes (Ram Scientific Inc, Yonkers, NY) were used to collect approximately 500 µl of blood from all consenting individuals. Tubes were stored in coolers until samples were processed the following day.

### Antigen Analysis

Antigenemia was assessed by two methods, ICT card (Binax, Portland, OR) and Og4C3 ELISA (TropBio, Townsville, Australia). Briefly, the day after blood collection, 100 µl of blood was added to the sample pad of an ICT card and read at 10 minutes according to manufacturer's instructions. Two lines in the viewing window of the card indicated a positive result [10]. Additional blood (60 µl per disk in 10 µl spots) was pipetted onto Trop Bio filter paper for shipment back to the CDC laboratory. Og4C3 antigen analysis was completed by diluting three blood spots per patient into 200 µl of dilution buffer and using the Og4C3-ELISA kit. Analyses were performed per manufacturer's instructions.

### Microfilaremia Analysis

For samples positive for antigen by ICT test, 60 µl of blood was pipetted on to microscope slides in three lines of 20 µl each. The slides were dehemaglobinized, fixed, stained with Giemsa stain and read by trained laboratory technicians at Hopital Ste. Croix.

### Antibody Analysis

Serum was eluted from filter paper blood spots to analyze the antibody response to the Bm14 antigen by ELISA [11]. Briefly, one blood spot per patient was eluted in 250 µl 0.05% PBS/Tween overnight at 4°C. Immulon ELISA plates were coated with recombinant Bm14 antigen at a concentration of 2 µg/ml in bicarbonate buffer and incubated overnight at 4°C. The following morning, plates were blocked for 1 hour at 4°C with 0.3% PBS/Tween. Eluates from the blood spots were added to the plates and incubated for 2 hours at room temperature. Biotinylated mouse anti-human IgG4 (Zymed, San Francisco, CA) was diluted 1∶1000 added to the plate and incubated for 1 hour at room temperature. The streptavidin-alkaline phosphatase (Invitrogen, Carlsbad, CA) was diluted 1∶1000 and again incubated for 1 hour at room temperature. Plates were washed in between each step with 0.05% PBS/Tween. Plates were visualized using 4-nitrophenyl phosphate disodium salt hexahydrate tablets (Sigma, St. Louis, MO) dissolved in 10% diethanolamine (DEA) and read on a spectrophotometer at 405 nm.

### Mosquito Collection and Analysis

Five CDC gravid traps were randomly placed around each community and the positions of the traps recorded with GPS. Mosquitoes were collected for five consecutive nights. Recently-fed vectors (blood-fed, gravid, or semi-gravid *Culex quinquefasciatus*) from each trap location were sorted and pooled in tubes of 1- 20 mosquitoes. Males and non-vector species were discarded. Mosquito pools were dried and shipped to the CDC. DNA was extracted from the mosquito pools at Smith College using a metal-bead vortex grinding technique [12] and a modified Qiagen DNeasy extraction column protocol [13],[14], followed by real-time PCR detection of the *W. bancrofti* LDR sequence as previously described [15]. Statistical analysis of the mosquito PCR results was performed using the PoolScreen v.2.02 program, designed to estimate the vector infection rate from pools of vectors [16].

### Statistical Analysis

Unless otherwise stated all statistical analysis was done using SAS version 9.1. The surveylogistic procedure was used to determine which possible transmission factors were significantly associated with filarial infection. This procedure was selected in order to properly weight the analysis based on the sampling design. The association between infection status and compliance was assessed by the chi-square test. A chi-square test was performed for each village and for the study area as a whole. Infection status was determined using a composite variable based on four tests for filarial infection. Global clustering of infected households, as well as of noncompliant households, was assessed by Cuzick and Edwards' nearest neighbor analysis at 2, 3, 4, and 5 nearest neighbors [17]. Households were defined as infected if at least one person in the household was positive for the various measures defined in the results. A Simes correction was used to control the overall type I error rate for multiple testing [18]. A Chi-square goodness-of-fit test was also used to compare the number of persons actually treated per household with the number of persons expected to be treated per household given the overall compliance rate in the village. The results of this test were used to determine the randomness of noncompliance within a village.

## Results

### Community Characteristics

A total of 564 people were surveyed by questionnaire. Of those who responded, a total of 455 (80.7%) people were tested for LF. People were not tested for LF because of refusal to give blood, low volumes of blood and loss to follow up. An average of 24 households per community were tested for LF of these, an average of seven households were originally selected as alternates. Reported age among tested individuals ranged from 3 to 95 years of age with the median age in the communities ranging from 18 to 25. Characteristics for the population tested for LF in each community are summarized in Table 1.

**Table 1:** Community characteristics of tested participants.

Community	N	Male	Female	Median Age	No. Households	Avg. number per household
Dufort	68	26	42	24	19	3.6
Guinebeau	96	36	60	22.5	27	3.6
Santo	72	25	47	23.5	23	3.1
Dampus	64	27	37	25	24	2.7
Leogane	87	26	61	18	24	3.6
Corail Lemaire	68	35	33	24.5	28	2.4

### Filarial Infection Prevalence

Filarial infection was assessed by four different methods; antigen detection by ICT card and Og4C3 ELISA; antibody detection by Bm14 ELISA; and microfilaria (MF) detection by microscopy. Blood films for microfilaremia were prepared only for individuals positive by ICT, so the MF prevalence represents a minimum value. Briefly, the minimum MF prevalence was 4.6% overall, with MF prevalences ranging from 0–7.8%. Overall antigen prevalence by ICT was 18.5%, with the highest prevalence in Dampus (32.8%) and the lowest prevalence in Corail Lemaire (2.9%). Overall antigen prevalence by Og4C3 test was 21.7%. Corail Lemaire again had the lowest antigen prevalence (5.1%), however the rank of ICT prevalence and Og4C3 prevalence were not identical for the other communities. Antibody prevalence, a measure of exposure to the parasite, was 47.0% overall. The highest antibody prevalence was found in Leogane at 72.4% and the lowest was in Corail Lemaire at 26.5%. Complete results for each community can by found in Table 2.

**Table 2:** Infection measures by community.

Community	Min. MF Prevalence	ICT Prevalence	Og4C3 Prevalence*	Bm14 Prevalence	Filarial DNA Rate
Dufort	**7.4%** (5/68)	**11.8%** (8/68)	**23.4%** (15/64)	**44.1%** (30/68)	**6.4%** (1075)
Guinebeau	**4.2%** (4/96)	**15.6%** (15/96)	**28.2%** (26/92	**35.4%** (34/96)	**11.5%** (1202)
Santo	**4.2%** (3/72)	**18.1%** (13/72)	**25%** (17/68)	**45.8%** (33/72)	**13.6%** (706)
Dampus	**7.8%** (5/64)	**32.8%** (21/64)	**26.9%** (14/52)	**56.3%** (36/64)	**27.9%** (1064)
Leogane	**4.6%** (4/87)	**28.7%** (25/87)	**19.1%** (16/84)	**72.4%** (63/87)	**26.7%** (1128)
Corail Lemaire	**0.0%** (0/68)	**2.9%** (2/68)	**5.1%** (3/59)	**26.5%** (18/68)	**N/A**
Overall	**4.6%** (21/455)	**18.5%** (84/455)	**21.7%** (91/419)	**47.0%** (214/455)	

*A smaller number of individuals were tested by Og4C3 due to low sample volumes.

**Figure 1:**
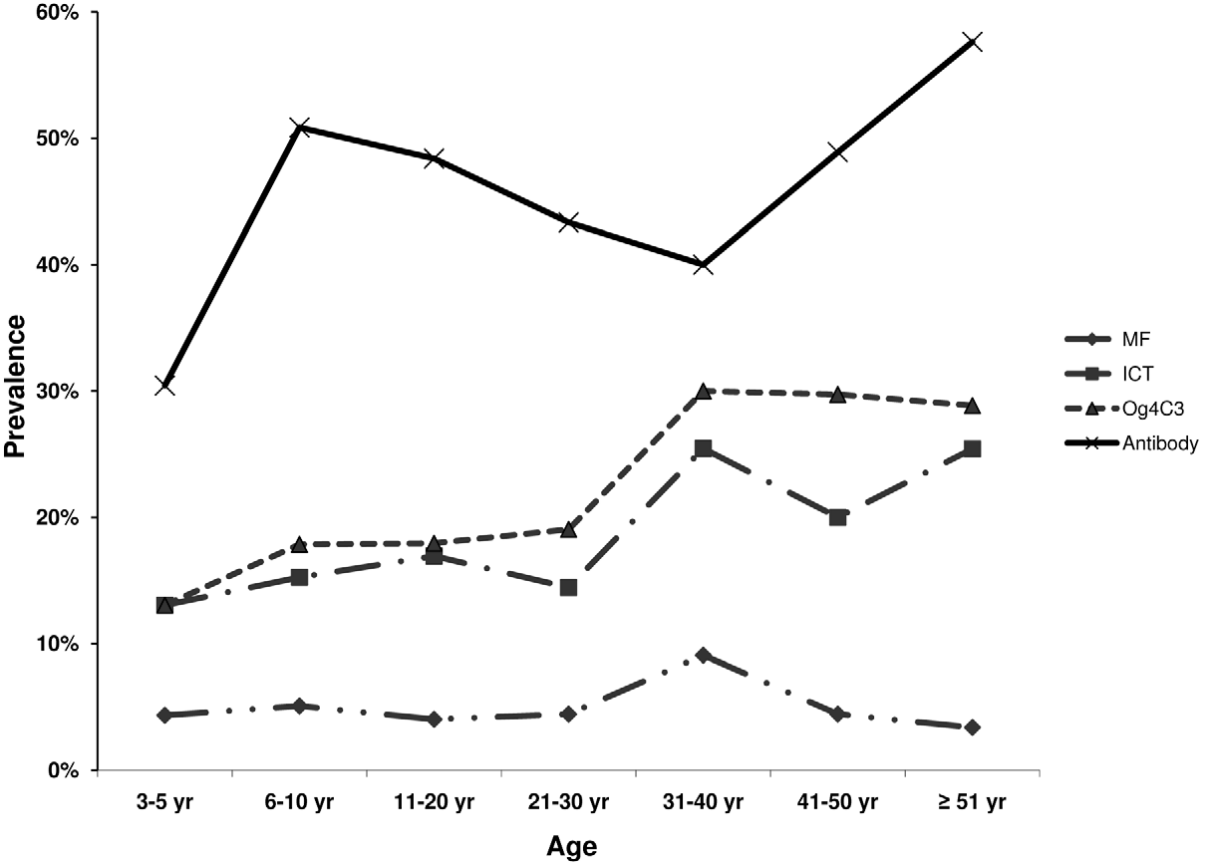
Age prevalence curves based on the various infection measures. Data are summarized across all communities. MF = microfilaremia ICT = filarial antigen detected by ICT card test. Og4C3  =  ELISA assay to detect filarial antigen Bm14 =  filarial antibody ELISA.

Age prevalence curves for all four infection measures are given in Figure 1. There is an overall trend of increasing prevalence for both antigen and antibody with age. The prevalence of MF was lower than baseline but not below the 1% cut-off required to stop MDA. Of note are the prevalences of infection by the different measures in the 3–5 year olds. Antigen prevalence in this age group was 13% by both antigen tests. Prevalence of MF was 4.3% and antibody prevalence was 30.4%.

### Filarial DNA Rates

Molecular xenomonitoring (MX) was performed using mosquitoes collected from each community except Corail Lemaire (logistical reasons). The average mosquito pool size was approximately 18 and the number of pools tested from each community ranged from 47 – 67. The filarial DNA rate was calculated for each community by the Poolscreen v.2.02 program (Table 3). Filarial DNA rates were high in all communities tested, ranging from 6.4% to 28%. Due to such high infection rates, and therefore high percentage of positive pools, the 95% confidence intervals were quite broad. The only significant difference in DNA rate was seen between Dufort (6.4%) and the two communities with the highest level of infection, Leogane (27%) and Dampus (28%). Despite this lack of statistical difference, the pattern of mosquito infection parallels the pattern of filarial infection with Dampus and Leogane being the two communities with the highest levels of both human and mosquito infection (Table 2).

**Table 3:** Molecular xenomonitoring (filarial DNA rates).

Community	# Mosq	# Pools	Mean Pool Size	# Pos. Pools	Max. Likelihood Est	95% Confidence Interval*
Dufort	1075	55	19.5	40	6.4%	4.3%–9.1%
Guinebeau	1202	67	17.9	58	11.5%	8.1%–16.0%
Santo	706	47	15.0	39	13.6%	8.8%–20.3%
Leogane	1128	62	18.2	60	26.7%	14.9%–59.4%
Dampus	1064	58	18.3	57	27.9%	15.1%–60.6.%
Total	5175	289	17.9	254		

*Calculated by the Likelihood Ratio Method.

### Noncompliance

**Figure 2:**
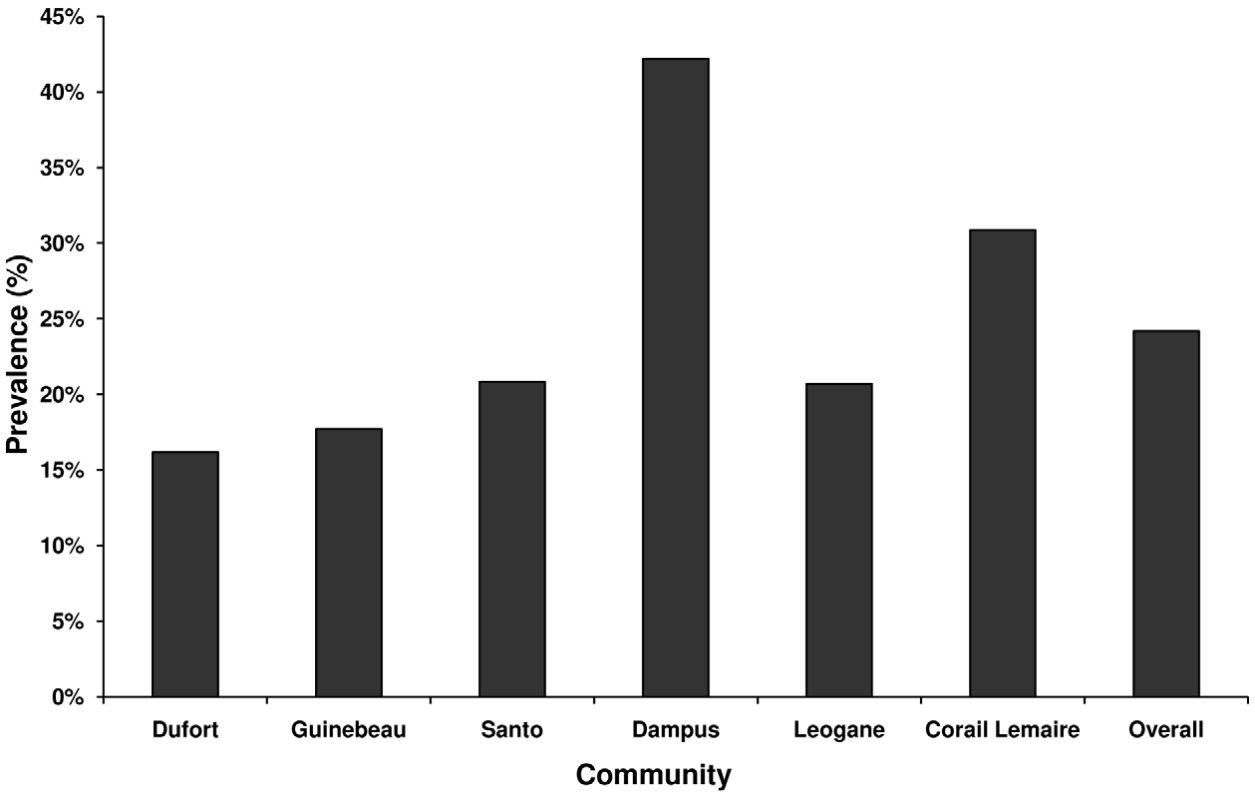
Prevalence of systematic noncompliant individuals by community. Individuals were considered noncompliant if they had reported never participating in MDA (i.e. systematic noncompliance).

Compliance status was assessed by asking individuals if they had ever taken a pill for filariasis. If they answered yes to that question, they were asked if they had taken a pill for filariasis during the more recent MDA or in prior MDAs. Of the 455 people who were tested for LF, 109 (24%), reported never having taken a pill for filariasis. One person did not respond to the question. Figure 2 illustrates the overall prevalence of systematic noncompliance (never taken a drug for LF) and prevalence of systematic noncompliance broken down by community. As with both human and filarial DNA rates, Dampus had the highest prevalence of systematic noncompliance at 42.2%. However, unlike human infection prevalence, the second highest prevalence of systematic noncompliance was in Corail Lemaire at 30.9%. This is contrast to the human and mosquito infection data, where Corail Lemaire had the lowest prevalence of both. The lowest prevalence of systematic noncompliance was found in Dufort with 16.2% corresponding with the lowest mosquito infection rate (6.4%).

Of the individuals who reported having taken a pill for filariasis 232 individuals (67.2%) reported being treated during the most recent MDA (2007) and 272 individuals (79%), reported having taking pills in 2005 or earlier. There was no MDA in Haiti during 2006.

**Figure 3:**
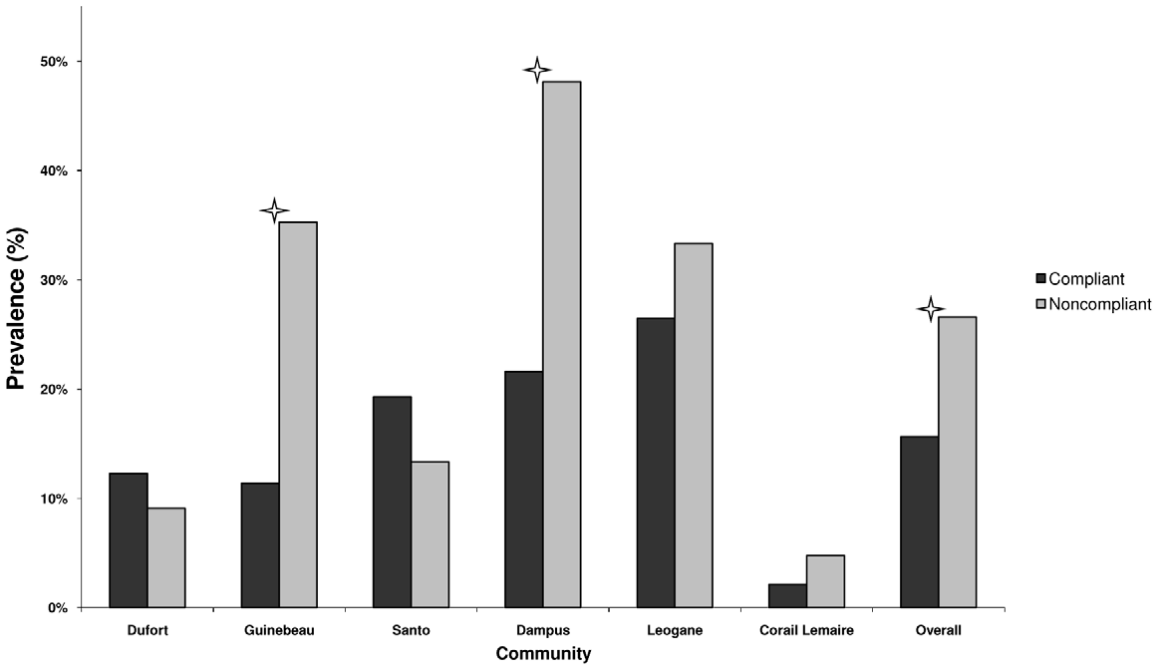
Prevalence of infection in compliant and noncompliant persons by community. ICT card test was used to determine infection prevalence. Noncompliant individuals were those who reported never participating in MDA. Compliant individuals were those who reported ever haven taken a drug for LF. The asterisk indicates a p-value <0.05. by Chi-square test.

In order to determine if noncompliance was associated with infection, we first examined the prevalence of infection determined by ICT card test in compliant and noncompliant individuals. Figure 3 shows the infection prevalence in compliant and noncompliant individuals. The overall infection prevalence was significantly higher in noncompliant individuals (26.6%) as compared with compliant individuals (15.7%) (p-value <0.05). Infection prevalence was also statistically higher in noncompliant individuals (35.3%) than in compliant individuals in Guinebeau (11.4%), (p<0.05) and in Dampus (48.1% among noncompliant persons vs. 21.6% among compliant persons, p-value <0.05). An increase in infection prevalence for noncompliant individuals was also seen in Corail Lemaire, and Leogane; however these differences were not statistically significant.

Those individuals who reported never having taken drugs for LF were asked their reason for not taking the pills. Table 4 is a compilation of the reasons individuals gave for not taking pills for LF. The number one reason was given as “don't know”. The second most common reason was “fear of side effects”, followed by “not in Leogane during the MDA”.

**Table 4:** Reasons for noncompliance.

Reason for Noncompliance	Frequency of Answer
Don't know	44
Fear of side effects	30
Not in Leogane during MDA	18
Taking other medication	7
Had side effects previously	5
Pregnant	2
Don't like to take pills	2
The pills are bad	1

A total of 109 noncompliant, LF tested individuals were used in the analysis. Multiple answers were not allowed.

### Factors Contributing to Continued Transmission

The factors examined in this study were migration to Leogane, travel from Leogane, knowledge of LF and noncompliance with the MDAs. In order to determine which variable(s) were significantly related to infection, univariate logistic regression analysis, controlling for village, was performed on the responses in the questionnaire. Of all the factors in this study, only compliance status was significantly related to infection as determined by ICT card test (p-value = 0.004, Table 5). Neither migration from areas outside of Leogane nor travel outside of Leogane were significantly related to infection status as only 3.5% of individuals tested had moved to Leogane and only 5.1% had traveled outside of Leogane in the past year.

**Table 5:** Potential factors for infection status.

Factor	P-value	Odds Ratio	95% CI
Sex	0.658	0.906	0.584–1.405
Age	0.089	1.010	0.998–1.022
Move to Leogane*	0.462	0.661	0.220–1.991
Travel**	0.206	1.764	0.732–4.250
Knowledge of LF	0.399	1.366	0.762–2.448
Knowledge of lymphedema	0.237	0.764	0.483–1.197
Knowledge of hydrocele	0.690	0.881	0.473–1.270
**Treatment status**	**0.004**	**1.971**	**1.244–3.121**

*A move was defined as anyone who had not lived in Leogane their entire life.

**An individual was considered to have traveled if they had been outside Leogane within the last year.

### Clustering and Spatial Analyses

Clustering of noncompliance by household was initially analyzed by Chi-square goodness-of-fit test; there was no evidence of clustering by household (data not shown).

Spatial analysis was done using the Cuzick and Edwards' nearest neighbors test. The number of antigen positive individuals per household ranged from 0 (59.4% of households) to 7 (0.7%). The number of non-compliant individuals per household ranged from 0 (53.9%) to 7 (0.7%). 40.6% of households had at least one antigen positive individual and 46.2% of households included at least one noncompliant individual. Table 6 gives the results of the test for various household attributes. Using ICT positivity as a measure of infection, there were more antigen positive households among the two, three, four and five nearest neighbors than would be expected by chance. These results indicate that from the two nearest neighbors up to the fifth nearest neighbors there was significant clustering of antigen positive households. For households with an antibody positive individual there was significant clustering for the two nearest neighbors. There was no clustering in households of noncompliance.

**Table 6:** Nearest neighbor analysis for spatial association.

Household Attribute	k = 2*	k = 3	k = 4	k = 5
Antigen positive by ICT test	0.042	0.005	0.004	0.014
Antibody positive by Bm14 test	0.003	0.104	0.205	0.357
Non-compliant	0.382	0.267	0.245	0.105

*Nearest neighbor analysis of spatial association by household. A household was considered positive if there is one positive individual in the household. k = the number of nearest neighbors. Significant test results indicate clustering of the tested variable. A Simes correction is used to control for the type I error rate for multiple testing. Highlighted results are significant at a Simes correction level using an alpha  = 0.05.

159 households out of the 180 households sampled were used in this analysis due participants refusing to be tested for LF. This led to households with incomplete data, which were excluded from the analysis.

## Discussion

In Haiti 7 rounds of MDA have been delivered to the commune of Leogane, with a missed round of MDA in 2006. By 2005 MF prevalence was below the <1% threshold that the WHO recommends for stopping MDA. However, subsequent studies in children suggested that transmission was ongoing despite the apparent success of the MDAs. The aim of this study was to determine the transmission status of LF in Leogane and to examine possible factors contributing to transmission in the area.

The levels of microfilaremia and antigenemia found in the communities in this study (with the exception of Corail Lemaire) indicate that transmission is still ongoing in Leogane. Only Corail Lemaire had MF prevalence below the <1% WHO threshold for stopping MDA. Antigen prevalence by both the ICT and Og4C3 test in children 3–5 years of age was above 10%. If transmission had been interrupted there should be little to no infection in this age group as they were born after the MDAs started. In addition to the high prevalence of microfilaremia and antigenemia in most of the communities surveyed, there was a high rate of infection in mosquitoes collected from those communities. The presence of a significant number of infected mosquitoes further supports the conclusion that transmission is ongoing. Although the MDA rounds have succeeded in lowering the overall prevalence of LF infection in Leogane, they have not succeeded in interrupting transmission of the parasite. Initial projections for the LF elimination predicted that 5–6 rounds of MDA would be sufficient to interrupt the transmission cycle of the parasite [19]. Since transmission was persistent in Haiti after more than the recommended rounds of MDA, migration, knowledge of LF, and noncompliance with MDA were considered as potential factors for ongoing transmission.

Of the factors examined, only systematic noncompliance was statistically associated with infection status. Noncompliance has been previously reported in Haiti. After 3 rounds of MDA in Leogane, 18.6% of adults surveyed had not participated in MDA [20]. Talbot et al found levels of noncompliance around 25% after 4 years of MDA [21]. The overall noncompliance rate in this study was very similar (24.2%). This noncompliance rate yields a compliance rate of around 76%, which is consistent with the WHO recommendation of >60–70% compliance for interruption of transmission. In Leogane, this level of compliance with MDA has not been adequate to interrupt transmission of LF.

These results suggest that a consistent proportion of the population has not been mobilized to participate in MDA. Social mobilization strategies employed by the program clearly are not reaching this segment of the population. It is important to acknowledge the limitations of questionnaire-based coverage surveys. It is possible that people do not remember taking the drug in previous years. Nonetheless, infection levels were significantly higher in noncompliant individuals as compared to compliant individuals, demonstrating a biologic correlate of the nonparticipation of the respondents in MDA. High rates of noncompliance maintain a reservoir of infection, which drives LF transmission.

Systematic noncompliance has been examined as a factor in MDA success in a number of different national elimination programs. In Egypt the noncompliance rate was very low (7.4%) and consequently, the program was able to successfully reduce infection levels to a point where transmission was probably interrupted [22]. Although residual infection rates were the highest in noncompliant individuals, the authors also observed a trend towards reduced infection rates in those individuals, indicating the possibility of a “herd treatment effect” as transmission levels decline [22]. These results are very encouraging for the Global Elimination Programme but they may not be transferable to Leogane where the baseline prevalence rates of MF and antigenemia were considerably higher than in Egypt. Also, the mode of drug distribution is different. In Egypt, health workers go door-to-door to distribute drugs, whereas in Haiti, community distribution points are used. A much higher compliance rate was seen with the door-to-door distribution in Egypt and that, combined with the lower baseline infection rates, led to the impressive results that were observed.

Other countries have experienced noncompliance rates more similar to that of Haiti. The compliance rates in India have been consistently low. In the southern state of Tamil Nadu compliance rates ranged between 46% and 64% [23],[24]. A 2005 study in Tamil Nadu found that only 30% of the study cohort complied with all six rounds of MDA and a study in Orissa state found 83% of the population had received the drug but only 49.5% consumed them [25],[26]. The picture is similar in other areas of the world. Recent studies in the Philippines and the Colombo district of Sri Lanka found noncompliance rates of 30% and 28% [27],[28]. However, it is important to distinguish between noncompliance for a single or several MDA rounds and systematic noncompliance (never taking a drug for LF). Sporadic noncompliance is problematic as it may reflect faults in the distribution system or education message of the MDA. However, if there is only sporadic noncompliance, all individuals in a community will have been treated at some point in time and the development of the “herd treatment effect” referred to by El-Setouhy et al will likely lead to an overall reduction in infection prevalence and interruption of transmission. Systematically noncompliant individuals continue to provide a reservoir of MF and perpetuate the transmission cycle. This is illustrated in our study with the high rates of systematic noncompliance, filarial infection and mosquito infection and the significant association between systematic noncompliance and filarial infection. Systematic noncompliance not only reflects possible weak points in the MDA program but threatens to undermine the program's goal of eliminating LF by interrupting transmission.

With similar rates of noncompliance in various countries, is there an underlying determinant or determinants that influence compliance with MDA? In the Philippines and India the perceived benefit of the MDA was associated with compliance. Knowledge of LF was found to be linked to MDA compliance in the Philippines and in Haiti [27],[29]. A KAP survey conducted in Haiti in 2000 as well as follow-up survey in 2004 found that women were more likely to be noncompliant [29]. This observation was also made in India [23]. In Haiti, the difference in compliance between the sexes was most likely due to the initial exclusion of women of child-bearing age from albendazole treatment during the first two rounds of MDA. This policy was reversed in 2002 and no discrepancy in compliance status was seen between males and females in our study. Other factors previously found to be associated with compliance status were ability to swallow pills and the perceived status of the interviewee in the community [21]. In the current study, the majority of noncompliant individuals cited “Don't know” as the reason for not participating in the MDA. The second highest response was fear of side effects (Table 4). While the side effects have reduced significantly in Leogane over the course of the MDAs [30], there seems to be residual concern based on the anticipation of side effects to the drugs. Equally as important are the “Don't know” respondents. Is this a proxy response for “Don't care” or is there some other determinant of compliance status that is not being captured in this or previous surveys? Averted cases of lymphedema and hydrocoele are hard to quantify and since there is little direct evidence that the MDA provides clinical relief for those chronic conditions, community members may not perceive any benefit to participating in MDA. Given that the age group with the highest noncompliance was 3–5 years of age, the de-worming effects of albendazole do not seem to be a major driver for parents to have their children participate in MDA. This could be due either to lack of exposure to messages regarding the de-worming properties of albendazole or a lack of understanding of these messages. Renewed health education efforts could provide incentive to participate by emphasizing the prevention of future lymphedema and hydrocele cases and the benefits of de-worming from albendazole. While health education messages were highly publicized at the beginning of the MDA cycle, the de-worming effects of albendazole were not emphasized. This added benefit could induce more individuals to participate, especially mothers and their children.

The variability of infection levels between communities (Table 2) emphasizes that infection is focal in nature, a reflection of poorly understood differences in mosquito habitat and density as well as host factors. There is previous evidence for spatial variation in LF infection. Ramzy et al reported non-uniform infection in LF [31]. A study done in 2001 in Papua New Guinea found micro-spatial heterogeneity in LF infection [32]. In Haiti, a 2003 study found spatial clustering of antigen positivity and IgG1 positivity [33]. The authors of that study concluded that the transmission dynamics of LF in Leogane may vary over as small a distance as tens of meters.

Using any of several definitions of infection status (e.g. antigen by ICT card or antibody positivity) we found evidence for clustering of filarial infection by the nearest neighbor analysis (Table 6). In contrast, noncompliance was not found to be spatially clustered. Why noncompliance would be statistically related to infection but not show the same spatial relationship as infection is unclear. There may be a spatial relationship to noncompliance that was not captured by this study, perhaps because of our sampling design, and further investigation of this relationship is warranted, specifically to determine the effect of a noncompliant individual on the infection status of his/her neighbors.

Although noncompliance was the only factor that was significantly related to infection in our study, it may not account for all of the transmission seen in Leogane. Initial infection prevalence, vector density, biting density and topography all play a role in infection. In a 2008 paper that used statistical methods to define the Risk of Infection Index (RII) based on community microfilariae load (CMFL) and vector density per man-hour (MHD), the authors concluded that transmission may continue in areas where MF prevalence is low but vector density is high [34]. In such situations it may be cost effective to use vector control, in contrast to areas of higher MF prevalence and low vector density where MDA may be the more cost effective tool [34]. The mosquito data collected in this study provides information about infection rates but not about vector and/or biting density. It was also not possible to make any conclusions about the vector-parasite relationship from this study. Investigations examining the effect of increases MF loads on vector survival have reported conflicting results and the exact relationship between *W. bancrofti* and its *Culex* vector is not clear [35]. Further studies are needed to examine vector issues in Haiti in order to determine the impact of vector ecologies on local transmission, and the nature of the parasite-vector relationship. This additional knowledge would aid in determining if vector control would be a cost effective measure to interrupt transmission there.

Population migration was not found to be a significant contributor to transmission in this study; however, this could be a consequence of the timing of the study. It is thought that the instability in Haiti between 2004 and 2005 led to a migration of individuals out of affected areas such as Port-au-Prince to less affected areas such as Leogane. Port-au-Prince has never undergone MDA, and these individuals could have represented a reservoir of infection. Since this study was undertaken several years after the unrest many of those individuals could have returned to their place of previous residence. If this scenario was true then these individuals would not be captured in the study. Thus, population migration may still be a factor in the ongoing transmission of LF in Leogane but was not reflected in this analysis.

An added factor contributing to transmission of LF is the missed round of MDA in Haiti in 2006. A study conducted in September 2007 by Won et al argued that this missed round of MDA was responsible for a rebound in infection to levels that were present in 2003 [36]. This rebound in infection rates underscores the importance of maintaining the MDA schedule and ensuring compliance in the population.

The LF elimination program in Leogane has been ongoing for eight years. The program has been successful in reducing MF and antigen rates from baseline levels over the course of seven rounds of MDA. Despite these achievements, and despite reaching the WHO benchmarks for success, there is ongoing transmission of LF in Leogane commune. It appears that one of the main contributors to this transmission is individuals who have never participated in the MDA. They may provide a pool of infection by which the mosquitoes become infected and the parasite is transmitted to others. The reasons for noncompliance are not completely clear. Also, noncompliance is one of many factors that could play a part in transmission. Other factors include vector density, a missed round of MDA, and the heterogeneity of transmission. New tools and approaches are needed in this environment in addition to the further studies recommended above. Increased health education and awareness campaigns may improve compliance. The addition of vector control methods such as insecticide treated bed nets could provide the extra push needed to stop transmission. It is also possible that DEC-fortified salt represents a programmatic alternative that should be re-visited. The incorporation of new tools should be investigated and implemented so that the program in Haiti can proceed toward elimination. The situation in Haiti is not dissimilar to that found in other parts of the world. Haiti can be used as a model for LF elimination in areas of high infection prevalence and high vector pressure. Understanding obstacles and solutions from the program in Haiti could be helpful for elimination programs in other countries.

## Supporting Information

Checklist S1 STROBE checklist.
(0.09 MB DOC)
